# Does the Length of Inter-Set Rest Periods Impact the Volume of Bench Pull Repetitions Completed before Surpassing Various Cut-Off Velocities?

**DOI:** 10.5114/jhk/188366

**Published:** 2024-12-06

**Authors:** Danica Janicijevic, Sergio Miras-Moreno, María Dolores Morenas-Aguilar, Amador García-Ramos

**Affiliations:** 1 Faculty of Sports Science, Ningbo University, Ningbo, China.; 2 Department of Radiology, Ningbo No. 2 Hospital, Ningbo, China.; 3 Department of Physical Education and Sport, Faculty of Sport Sciences, University of Granada, Granada, Spain.; 4 Department of Sports Sciences and Physical Conditioning, Faculty of Education, Universidad Catolica de la Santísima Concepcion, Concepción, Chile.

**Keywords:** fatigue, resistance training, velocity-based training, linear position transducer

## Abstract

This study aimed to determine the optimal inter-set rest periods that would maximize the number of repetitions completed before surpassing various cut-off velocities (COVs) during the prone bench pull exercise. Twenty-three physically active individuals, 15 men and 8 women, participated in six random testing sessions. Each session included four sets of the prone bench pull exercise performed with maximum intent on a Smith machine at 75% of the one-repetition maximum (1RM). The length of the inter-set rest interval (1 [R1], 3 [R3], and 5 [R5] min) and COV used (0.65 m•s^−1^ [COV_0.65_] and 0.55 m•s^−1^ [COV_0.55_]) varied between sessions. Longer inter-set rest periods led to a higher volume of repetitions (R5 > R3 > R1), whereas the differences between the rest protocols were larger for COV_0.55_ (R1: 28.4 ± 6.0 repetitions; R3: 36.4 ± 9.4 repetitions; R5: 41.1 ± 11.4 repetitions) compared to COV_0.65_ (R1: 24.2 ± 7.3 repetitions; R3: 25.4 ± 10.1 repetitions; R5: 28.3 ± 9.7 repetitions). Increasing the number of sets negatively impacted the number of completed repetitions for R1 using both COV_0.65_ and COV_0.55_, as well as for R3 using COV_0.55_. The fastest velocity of the set (MV_fastest_) did not differ between the inter-set rest protocols for COV_0.65_, while for COV_0.55_, R3 and R5 provided a greater MV_fastest_ than R1 for sets 2–4. These findings suggest that the duration of inter-set rest periods is an important factor to consider when aiming to maximize mechanical performance across multiple sets of the prone bench pull exercise.

## Introduction

Resistance training (RT) has been shown to provide numerous benefits, including improvements in muscular strength and endurance, bone density, body composition, metabolic health, and overall physical function (ACSM, 2009; Erickson et al., 2019). Developing muscular strength through RT has also been demonstrated to improve overall athletic performance and decrease the probability of injury (Faigenbaum and Myer, 2010; Weakley et al., 2023). The manipulation of variables such as the exercise type and order, exercise intensity (i.e., the load lifted with respect to the maximal dynamic strength capacity [i.e., the 1-repetition maximum; 1RM]), volume of repetitions, duration of the intra- and inter-set rest periods, and intended lifting velocity is well known to modulate RT-induced adaptations (Bird et al., 2005; Kraemer and Ratamess, 2004). Recognizing the intricate relationship of acute RT variables is critical, as altering one can have a direct impact on another, emphasizing the need of a well-designed RT program that balances these variables for optimal adaptations. For example, the volume of repetitions that can be completed is inversely related with the load lifted (Bird et al., 2005; Kraemer and Ratamess, 2004), whereas the exercise, range of motion and a lifting tempo also influence the number of repetitions that can be completed to failure when employing the same relative intensity (%1RM) (Tsoukos et al., 2024; Wilk et al., 2018, 2018a).

The concept of training to failure, which entails performing repetitions until it is impossible to complete another one with proper technique, has been a topic of interest in RT research (Akcan et al., 2024; Davies et al., 2016; Grgic et al., 2022; Lopez et al., 2021). While many studies have investigated how various factors (e.g., exercise choice, exercise intensity, inter-set rest periods, or the lifting tempo) affect training volume using sets to failure (Grgic et al., 2022; Lopez et al., 2021), this approach does not fully reflect most athletes' training routines. Indeed, it is generally accepted that training to failure can cause excessive fatigue, resulting in longer recovery times between sessions and negatively impacting adaptations in overall athletic performance (Davies et al., 2016; Grgic et al., 2022). Therefore, it appears more relevant to investigate the effects of the previously mentioned factors on mechanical performance during RT sessions that do not include sets to failure (Gutiérrez-Flores et al., 2024). This line of research could be useful in devising tactics for increasing the number of repetitions that can be completed before surpassing various cut-off velocities (COVs). Cut-off velocities, alternatively referred to as velocity stop values, serve to conclude a set when the average concentric velocity of a repetition falls below a predetermined velocity value.

There is substantial evidence that lengthening inter-set rest periods increases the number of repetitions that can be completed until failure (De Salles et al., 2009; Gonzalez, 2016; Grgic et al., 2017, 2018). These findings are not surprising given that longer rest intervals allow for superior replenishment of energy substrates and removal of metabolic waste products, resulting in better performance in subsequent sets (Kraemer and Ratamess, 2005; Ratamess et al., 2007). However, it is well recognized that the need for energy substrates and the creation of metabolic waste products are also affected by the proximity to failure (Davies et al., 2016). As a result, shorter rest periods may be required as the sets are terminated farther from failure (i.e., utilizing greater COV). When completing three sets of five repetitions against the 10RM load during the squat and bench press exercises, it has been demonstrated that 3 and 5 min of inter-set rest allow for greater velocity performance than 1 min of inter-set rest (González-Hernández et al., 2023). However, it is unknown whether the length of inter-set rest periods also impacts the volume of repetitions that can be completed before reaching different COV. When short inter-set rest periods are used, it is also possible that the fastest mean velocity of the set (MV_fastest_) is progressively reduced over the RT session, which can potentially impact the computations of two variables commonly used in practice: mean velocity decline (MVD) and mean velocity maintenance (MVM) (Jukic et al., 2023; Tufano et al., 2016). MVD, computed using the fastest MV of each specific set, has been employed to prescribe the volume of repetitions in RT sessions comprising multiple sets (Pareja-Blanco et al., 2017, 2020a, 2020b), whereas MVM is used for testing the ability to maintain high velocity output during a set of multiple repetitions (Tufano et al., 2016).

To gain insight into these issues, the present investigation involved subjects executing four sets of the prone bench pull exercise on distinct occasions until they surpassed two COVs (0.65 m•s^−1^ [COV_0.65_] and 0.55 m•s^−1^ [COV_0.55_]), with inter-set rest periods of 1 min [R1], 3 min [R3], and 5 min [R5]. The objective of this study was threefold: (i) to assess the impact of inter-set rest periods with varying duration (R1, R3, and R5) on the number of repetitions performed before surpassing COV_0.65_ and COV_0.55_, (ii) to describe the changes in the fastest mean velocity of the set (MV_fastest_) during RT sessions that varied in the duration of inter-set rest periods and COVs, and (iii) to examine the differences in MVD and MVM when using for their computation the MV_fastest_ of each set (MVD_individual_ and MVM_individual_), MV_fastest_ of the first set (MVD_first_ and MVM_first_), and MV_fastest_ of the entire training session (MVD_session_ and MVM_session_). It was hypothesized that (i) longer inter-set rest periods (R5 > R3 > R1) would lead to a higher volume of repetitions completed for both COV_0.65_ and COV_0.55_, (ii) the MV_fastest_ would decrease as the number of sets increased (set 1 > set 2 > set 3 > set 4) with a more pronounced decrement for shorter inter-set rest periods (R1 > R3 > R5), and (iii) the reference repetition would impact the magnitude of both MVD and MVM, with more positive values (i.e., reflecting a higher mechanical performance [MVM] or lower fatigue [MVD]) obtained when using the MV_fastest_ of each set, followed by the MV_fastest_ of the first set, and finally the MV_fastest_ of the entire training session.

## Methods

### Participants

Considering an effect size of f = 0.25 (a moderate effect size), an α error probability of 0.05, a power of 0.80, 3 groups, 6 measurements, and assuming a correlation among repeated measures of 0.5, the sample size calculation indicated that a total of 21 subjects would be sufficient for detecting the postulated effects (G*Power software, version 3.1.9.6). Therefore, 23 healthy individuals with a regular physical exercise routine participated in this study. The group consisted of 15 men (age: 23.7 ± 4.3 years; body mass: 79.7 ± 10.7 kg; body height: 1.79 ± 0.08 m; prone bench pull 1RM: 86.0 ± 11.6 kg [1RM relative to body mass: 1.08 ± 0.15]) and 8 women (age: 25.9 ± 8.7 years; body mass: 61.6 ± 6.0 kg; body height: 1.64 ± 0.04 m; prone bench pull 1RM: 49.7 ± 6.1 kg [1RM relative to body mass: 0.81 ± 0.10]) (data presented as means ± standard deviations [SD]). All participants had prior experience with the prone bench pull exercise and did not have any physical limitations that could affect the study results. They were informed about the study's purposes and procedures and gave their consent by signing an informed consent form before participating. The study protocol followed the principles outlined in the Declaration of Helsinki and was approved by the ethics committee of the University of Granada (protocol code: 2046/CEIH/2021; date of approval: 19 March 2021).

### Design

A crossover design was used to examine how varying the length of inter-set rest periods and proximity to muscular failure during the prone bench pull exercise can influence both maximal velocity production and maximal velocity maintenance capacities. Participants completed six testing sessions, with each session separated by a recovery period of 48–96 h. Due to the participants' previous experience with the testing protocols and familiarity with the prone bench pull exercise from their involvement in similar research conducted by our research group, a dedicated familiarization session for this study was deemed unnecessary. The six testing sessions, which were applied in random order, varied in two aspects: the duration of the inter-set rest period (1 min [R1], 3 min [R3], and 5 min [R5]) and the COV applied (0.65 m•s^−1^ [COV_0.65_] and 0.55 m•s^−1^ [COV_0.55_]). Note that when the MV declines below 0.55 m•s^−1^ subjects are unlikely to perform any more successful repetitions, while when the MV is 0.65 m•s^−1^ subjects can complete on average 2–3 more repetitions before reaching failure (Pérez-Castilla et al., 2023). In each session participants performed four sets of the bench pull exercise using a Smith machine at maximum intended velocity. The experimental sessions took place in the university research laboratory, and each participant performed their sessions at the same time of the day to prevent diurnal variations in strength performance.

### Testing Procedures

A general warm-up that included running and upper-body joint mobilization exercises was completed followed by a specific warm-up consisting of an incremental loading test using the Smith machine prone bench pull exercise. The initial load was 20 kg, which was gradually raised in 10-kg increments until the MV was less than 0.80 m•s^−1^. Then, the load was gradually increased in stages of 5 to 1 kg until the 1RM was reached (MV ~0.47 m•s^−1^). Two repetitions with light to moderate loads (MV > 0.80 m•s^−1^) and one repetition with a greater load (MV < 0.80 m•s^−1^) were performed. The inter-set recovery period for light-moderate loads was set at 3 min and 5 min for heavier loads.

Participants were allowed 10 min to rest between the determination of 1RM and the beginning of the first set of the training session. In each session, participants performed at maximum intended velocity four sets against the 75%1RM during the Smith machine prone bench pull exercise. The length of the inter-set rest periods (R1, R3, and R5) and the COV used (COV_0.65_ and COV_0.55_) varied between the six testing sessions. Participants were given real-time MV feedback after performing each repetition, and they were encouraged to maximize their MV output in each repetition (Jiménez-Alonso et al., 2022). Participants were advised to stop the set by an experienced researcher when one repetition failed to be greater than the COV stipulated for that session. Detailed explanation of the prone bench pull execution technique has been provided elsewhere (Miras-Moreno et al., 2022).

### Measurement Equipment and Data Analysis

A validated linear position transducer (GymAware RS, Kinetic Performance Technologies, Canberra, Australia) was vertically mounted to the Smith machine's barbell (Multipower Fitness Line, Peroga, Murcia, Spain) and provided the MV of each repetition (Weakley et al., 2021). This study considered as dependent variables (i) the maximum number of repetitions completed before exceeding different COVs (MNR), (ii) fastest MV of the set (MV_fastest_), (iii) MV of the last repetition of the set (MV_last_), (iv) mean velocity decrement (MVD [%] = [MV_last_ – MV_fastest_] / MV_fastest_ × 100), and (v) mean velocity maintenance (MVM [%] = MV_set_ × 100 / MV_fastest_). The MV_set_ represents the average MV of all repetitions completed in the set. Three reference repetitions were considered for computing MVD and MVM: (i) the MV_fastest_ of each particular set (MVD_individual_ and MVM_individual_), (ii) the MV_fastest_ of the first set (MVD_first_ and MVM_first_), and (iii) the MV_fastest_ of the training session (MVD_session_ and MVM_session_).

### Statistical Analyses

Descriptive data are presented as means and standard deviations. A three-way repeated-measures analysis of variance (ANOVA) with Bonferroni post hoc corrections (*rest* [R1 *vs*. R3 *vs*. R5], *set* [set 1 *vs*. set 2 *vs*. set 3 *vs*. set 4], and *COV* [COV_0.65_ *vs*. COV_0.65_]) was applied to MNR, MV_fastest_, and MV_last_. The sex factor was not considered in the ANOVAs because it failed to reveal any significant main effect or interaction for any dependent variable. A repeated-measures ANOVA with Bonferroni post hoc corrections (*rest* [R1 *vs*. R3 *vs*. R5], *set* [set 1 *vs*. set 2 *vs*. set 3 *vs*. set 4], and *reference repetition* [individual set *vs*. first set *vs*. fastest set]) was also applied to the MVD and MVM computed during the high-fatigue RT session (i.e., COV_0.55_). The Greenhouse-Geisser correction was used when the assumption of the homogeneity of variance was violated according to the Levene's tests (*p* < 0.05). The set number that provided the fastest repetition of the training session was also indicated for descriptive purposes. All statistical analyses were performed using SPSS software version 22.0 (SPSS Inc., Chicago, IL, USA) and statistical significance was set at an alpha level of 0.05.

## Results

### MNR, MV_fastest_, and MV_last_

**Figure 1:**
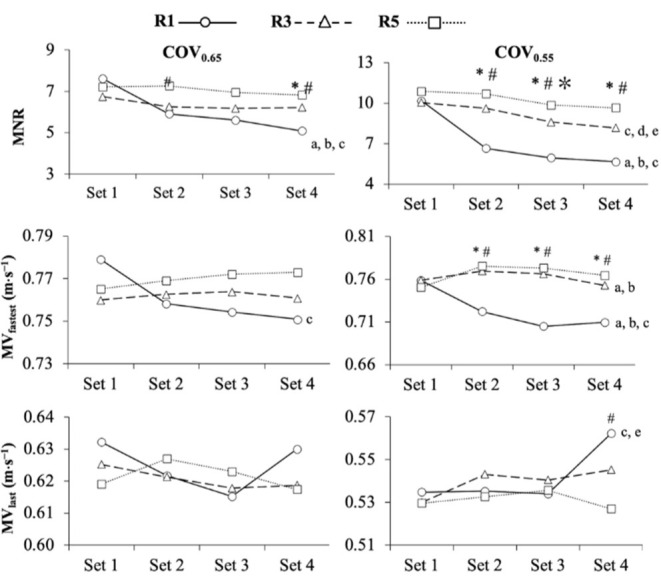
Comparison of the maximum number of repetitions completed (MNR; upper-panels), mean velocity of the fastest repetition (MV_fastest_; middle-panels), and mean velocity of the last repetition (MV_last_; lower-panels) among the different inter-set rest periods and sets using cut-off velocities of 0.65 m•s^−1^ (COV_0.65_; left panels) and 0.55 m•s^−1^ (COV_0.55_; right panels). R1, 1 min of inter-set rest; R3, 3 min of inter-set rest; R5, 5 min of inter-set rest; *, significant differences between R1 and R3; #, significant differences between R1 and R5; ✼, significant differences between R3 and R5; a, significant differences between set 1 and set 2; b, significant differences between set 1 and set 3; c, significant differences between set 1 and set 4; d, significant differences between set 2 and set 3; e, significant differences between set 2 and set 4

ANOVA results and pairwise comparisons for MNR, MV_fastest_, and MV_last_ are depicted in Table 1 and Figure 1, respectively. The ANOVA conducted on MNR revealed a significant main effect of rest (R5 > R3 > R1; *p* < 0.001), COV (COV_0.55_ > COV_0.65_; *p* < 0.001), and set (set 1 > set 2 > set 3 > set 4; *p* < 0.001). The triple interaction (rest × COV × set) was not significant (*p* = 0.295), but the remaining interactions reached statistical significance. The rest × COV interaction (*p* = 0.002) was induced by the fact that the differences between the inter-set rest protocols were larger for COV_0.55_ than COV_0.65_. The rest × set interaction (*p* < 0.001) was caused because there were no significant differences between the inter-set rest protocols in the first training set, but the differences were identified in the second set and remained consistent across the sets. The COV × set interaction (*p* < 0.001) resulted from the fact that the differences between the sets were greater for COV_0.55_ than for COV_0.65_.

**Table 1:** Three-way repeated-measures analysis of variance (ANOVA) comparing MNR, MV_fastest_, and MV_last_ among the inter-set rest periods, cut-off velocities, and the set number.

Variable	Rest	COV	Set number				ANOVA
			Set 1	Set 2	Set 3	Set 4	
MNR	R1	COV_0.65_	7.6 (2.8)	5.9 (2.3)	5.6 (2.0)	5.1 (1.5)	Rest: *p* < 0.001 MVT: *p* < 0.001 Set: *p* < 0.001 Rest×MVT: *p* = 0.002 Rest×Set: *p* < 0.001 MVT×Set: *p* < 0.001 Rest×MVT×Set: *p* = 0.295
		COV_0.55_	10.2 (2.9)	6.7 (1.4)	6.0 (1.8)	5.7 (1.7)	
	R3	COV_0.65_	6.7 (3.0)	6.3 (3.2)	6.2 (2.5)	6.2 (2.7)	
		COV_0.55_	10.0 (3.2)	9.6 (2.7)	8.6 (2.3)	8.2 (2.5)	
	R5	COV_0.65_	7.2 (2.9)	7.3 (2.7)	7.0 (2.7)	6.8 (2.4)	
		COV_0.55_	10.9 (3.1)	10.7 (3.6)	9.9 (2.5)	9.7 (3.7)	
MV_fastest_ (m•s^−1^)	R1	COV_0.65_	0.78 (0.05)	0.76 (0.04)	0.75 (0.04)	0.75 (0.04)	Rest: *p* = 0.001 MVT: *p* = 0.003 Set: *p* = 0.054 Rest×MVT: *p* = 0.002 Rest×Set: *p* < 0.001 MVT×Set: *p* = 0.214 Rest×MVT×Set: *p* = 0.041
		COV_0.55_	0.76 (0.06)	0.72 (0.05)	0.70 (0.04)	0.71 (0.04)	
	R3	COV_0.65_	0.76 (0.05)	0.76 (0.06)	0.76 (0.05)	0.76 (0.06)	
		COV_0.55_	0.76 (0.05)	0.77 (0.05)	0.77 (0.05)	0.75 (0.06)	
	R5	COV_0.65_	0.77 (0.06)	0.77 (0.06)	0.77 (0.06)	0.77 (0.05)	
		COV_0.55_	0.75 (0.05)	0.78 (0.06)	0.77 (0.06)	0.76 (0.07)	
MV_last_ (m•s^−1^)	R1	COV_0.65_	0.63 (0.02)	0.62 (0.02)	0.62 (0.03)	0.63 (0.03)	Rest: *p* = 0.263 MVT: *p* < 0.001 Set: *p* = 0.369 Rest×MVT: *p* = 0.520 Rest×Set: *p* = 0.045 MVT×Set: *p* = 0.118 Rest×MVT×Set: *p* = 0.569
		COV_0.55_	0.53 (0.04)	0.54 (0.04)	0.53 (0.06)	0.56 (0.04)	
	R3	COV_0.65_	0.63 (0.02)	0.62 (0.02)	0.62 (0.02)	0.62 (0.02)	
		COV_0.55_	0.53 (0.04)	0.54 (0.02)	0.54 (0.04)	0.55 (0.04)	
	R5	COV_0.65_	0.62 (0.02)	0.63 (0.02)	0.62 (0.01)	0.62 (0.02)	
		COV_0.55_	0.53 (0.06)	0.53 (0.02)	0.54 (0.03)	0.53 (0.04)	

MNR, number of repetitions completed; MV, mean velocity; COV, cut-off velocity; R1, 1 min of inter-set rest; R3, 3 min of inter-set rest; R5, 5 min of inter-set rest. Bold letters indicate p values lower than 0.05. Descriptive data are presented as means and standard deviations

The ANOVA conducted on MV_fastest_ revealed a significant main effect of rest (R5 > R3 > R1; *p* = 0.001) and COV (COV_0.65_ > COV_0.55_; *p* = 0.003), but the main effect of set was not significant (*p* = 0.054). The COV × set interaction was not significant (*p* = 0.214), but the remaining interactions reached statistical significance. The rest × COV interaction (*p* = 0.002) arose because the differences between the inter-set rest protocols were greater for COV_0.55_ than for COV_0.65_. The rest × set interaction (*p* < 0.001) was a result of no significant differences between the inter-set rest protocols in the first training set, but the differences were identified in the second set and remained stable or slightly increased across the sets. Finally, the rest × COV × set interaction (*p* < 0.001) resulted from the fact that the differences between the inter-set rest protocols were increased with the increment in the number of sets for COV_0.65_ but not for COV_0.55_.

The ANOVA conducted on MV_last_ revealed a significant main effect of COV (COV_0.65_ > COV_0.55_; *p* < 0.001) and a significant rest × set interaction (*p* = 0.045) because MV_last_ was greater in set 4 for R1, yet no significant differences between the inter-set rest protocols were observed for the remaining sets. Other main effects and interactions failed to reach statistical significance (*p* > 0.05).

### MVD and MVM

**Figure 2:**
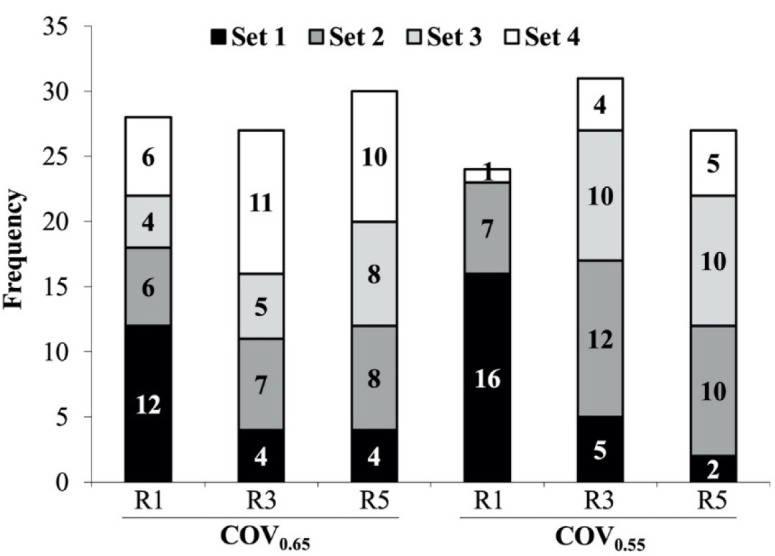
Number of times each set provided the fastest repetition of the training session. More than one set was counted when the fastest velocity was the same (two decimal place sensitivity) for them. COV, cut-off velocity; R1, 1 min of inter-set rest; R3, 3 min of inter-set rest; R5, 5 min of inter-set rest

**Figure 3:**
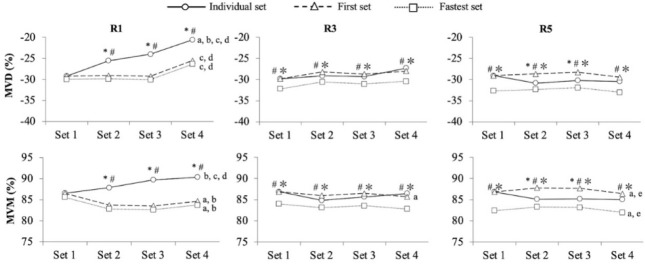
Comparison of mean velocity decline (MVD; upper-panels) and mean velocity maintenance (MVM; lower-panels) computed using different reference repetitions (individual set, first set, and fastest set) across four sets separated by 1 (R1; left-panels), 3 (R3; middle-panels), and 5 (R5, right-panels) min of rest. *, significant differences between the individual set and the first set; #, significant differences between the individual set and the fastest set; ✼, significant differences between the first set and the fastest set; a, significant differences between set 1 and set 2; b, significant differences between set 1 and set 3; c, significant differences between set 1 and set 4; d, significant differences between set 2 and set 4; e, significant differences between set 3 and set 4

The fastest repetition was almost always obtained in the first set of the training session for the R1 protocol, but it was more often obtained in following sets (sets 2–4) for the R3 and R5 protocols (Figure 2). The ANOVA results and pairwise comparisons for MVD and MVM are depicted in Table 2 and Figure 3, respectively. The ANOVA conducted on MVD revealed a significant main effect of rest (R1 < R3 = R5; *p* = 0.039), reference repetition (MVD_individual_ < MVD_first_ < MVD_session_; *p* < 0.001), and set (set 4 < set 1 = set 2 = set 3; *p* = 0.048). All the interactions reached statistical significance.

**Table 2:** Three-way repeated-measures analysis of variance (ANOVA) comparing the MVD and MVM obtained when using a cut-off velocity of 0.55 m•s^−1^ among the inter-set rest periods, reference repetitions, and the set number.

Variable	Rest	Reference repetition	Set number				ANOVA
			Set 1	Set 2	Set 3	Set 4	
MVD (%)	R1	Individual set	29.2 (6.3)	25.6 (7.1)	24.0 (9.5)	20.6 (6.4)	Rest: *p* = 0.039 Rep: *p* < 0.001 Set: *p* = 0.048 Rest×Rep: *p* < 0.001 Rest×Set: *p* = 0.045 Rep×Set: *p* = 0.004 Rest×Rep×Set: *p* < 0.001
		First set	29.2 (6.3)	29.1 (6.6)	29.3 (9.1)	25.6 (7.3)	
		Fastest set	30.0 (5.9)	29.9 (6.2)	30.1 (8.8)	26.4 (7.0)	
	R3	Individual set	29.8 (7.1)	29.1 (5.2)	29.3 (5.1)	27.3 (5.8)	
		First set	29.8 (7.1)	28.2 (4.5)	28.7 (4.4)	28.0 (5.4)	
		Fastest set	32.2 (6.6)	30.6 (5.1)	31.1 (4.7)	30.4 (4.9)	
	R5	Individual set	29.1 (9.0)	30.9 (6.5)	30.2 (7.3)	30.5 (8.9)	
		First set	29.1 (9.0)	28.7 (6.1)	28.3 (6.6)	29.5 (7.4)	
		Fastest set	32.7 (8.7)	32.3 (6.0)	31.9 (6.7)	33.0 (7.5)	
MVM (%)	R1	Individual set	86.5 (2.8)	87.9 (3.7)	89.7 (3.7)	90.4 (2.8)	Rest: *p* = 0.515 Rep: *p* < 0.001 Set: *p* = 0.058 Rest×Rep: *p* < 0.001 Rest×Set: *p* = 0.019 Rep×Set: *p* < 0.001 Rest×Rep×Set: *p* < 0.001
		First set	86.5 (2.8)	83.7 (4.5)	83.5 (4.7)	84.6 (4.4)	
		Fastest set	85.6 (2.9)	82.8 (3.7)	82.6 (4.5)	83.7 (4.0)	
	R3	Individual set	86.9 (2.4)	84.8 (2.9)	85.7 (2.7)	86.5 (3.0)	
		First set	86.9 (2.4)	86.0 (2.8)	86.5 (3.3)	85.7 (3.5)	
		Fastest set	84.0 (3.1)	83.1 (3.1)	83.6 (2.9)	82.8 (2.7)	
	R5	Individual set	86.9 (3.0)	85.1 (3.2)	85.2 (3.0)	85.0 (4.1)	
		First set	86.9 (3.0)	87.8 (3.3)	87.7 (3.5)	86.4 (3.3)	
		Fastest set	82.4 (3.3)	83.3 (2.9)	83.2 (3.0)	82.0 (2.7)	

MVD, mean velocity decline; MVM, mean velocity maintenance; R1, 1 min of inter-set rest; R3, 3 min of inter-set rest; R5, 5 min of inter-set rest. Bold letters indicate p values lower than 0.05. Descriptive data are presented as means and standard deviations

The rest × reference repetition interaction (*p* < 0.001) was induced by the fact that the differences between the reference repetitions were greater for R1 compared to R3 and R5. The rest × set interaction (*p* = 0.045) was caused because MVD remained stable across the sets for R3 and 5, but it increased with the succession of sets for R1. The reference repetition × set interaction (*p* = 0.004) resulted from the fact that the differences between the reference repetitions were lower at set 1 compared to the remaining sets (set 2–4), while the triple interaction (*p* < 0.001) was caused because the lower differences at set 1 compared to the remaining sets between the reference repetitions were accentuated for the R1 protocol.

The ANOVA conducted on MVM revealed a significant main effect of the reference repetition (MVM_individual_ > MVM_first_ > MVM_session_; *p* < 0.001), but the main effects of rest (*p* = 0.515) and set (*p* = 0.058) were not significant. All the interactions reached statistical significance. The rest × reference repetition interaction (*p* < 0.001) was induced by the fact that the differences between the reference repetitions were greater for R1 compared to R3 and R5. The rest × set interaction (*p* = 0.019) was caused by MVM remaining steady across sets for R3 and R5, but it increased with the succession of sets for R1. The reference repetition × set interaction (*p* < 0.001) resulted from the fact that the differences between the reference repetitions were lower at set 1 compared to sets 2–4. The triple interaction (*p* < 0.001) was caused because the lower differences at set 1 compared to the remaining sets between the reference repetitions were accentuated for the R1 protocol.

## Discussion

This study investigated the effects of different inter-set rest periods on mechanical performance (MNR, MV_fastest_, MVD and MVM) during sets of the bench pull exercise performed with moderate (COV_0.65_) and high (COV_0.55_) levels of fatigue. Longer inter-set rest periods resulted in a greater MNR (R5 > R3 > R1). Irrespective of the proximity to failure, R1 always resulted in a decline in the MNR as the number of sets increased, but R5 produced a consistent MNR across the sets. Regarding R3, the proximity to failure impacted the changes in the MNR across the sets, decreasing using COV_0.55_, but remaining stable using COV_0.65_. The differences between the inter-set rest protocols for MV_fastest_ were also affected by the proximity to failure. For COV_0.65_, no significant differences were observed, while for COV_0.55_, R3 and R5 provided greater MV_fastest_ than R1 for sets 2–4. MVD and MVM were affected by the reference repetition used for their computation, being recommended to use the MV_fastest_ from the first set or from the entire training session instead of the MV_fastest_ of each specific set to avoid overestimation of mechanical performance as MV_fastest_ decreased due to fatigue with an increase in the number of sets. These findings demonstrate that inter-set rest period length is an important consideration when seeking to maximize mechanical performance throughout multiple sets of the bench pull exercise and that more than 3-min recovery can be required to maximize performance.

Our initial hypothesis was substantiated, as longer inter-set rest periods were found to facilitate a greater MNR, regardless of the proximity to failure. These findings align with previous research demonstrating a higher number of repetitions performed to failure during the bent-over row exercise at 75% of 1RM when employing inter-set rest periods of 2 min compared to the duration of 1 and 1.5 min (Azzeme et al., 2020). Consistent findings have been reported across various exercises when sets are performed to failure (De Salles et al., 2009). Although the present study expands on the performance-enhancing effects of longer inter-set rest periods on the MNR to sets not leading to failure, the proximity to failure was shown to influence the MNR throughout the multiple sets of the RT session. Specifically, R1 always resulted in a decline in the MNR as the number of sets increased, R3 resulted in a decline in the MNR as the number of sets increased using a high (COV_0.55_) but not a moderate (COV_0.65_) level of fatigue, and R5 produced a consistent MNR across all four sets. These results are not surprising considering the extensive body of evidence demonstrating that higher levels of fatigue (i.e., resulting in greater velocity losses), regardless of whether sets are performed to failure or not, consistently result in increased metabolic markers such as lactate or ammonia (Jukic et al., 2023; Sánchez-Medina and González-Badillo, 2011; Weakley et al., 2020a). Therefore, the minimal duration of inter-set rest periods to maintain consistent mechanical performance across multiple sets appears to be contingent upon the level of fatigue, with greater proximity to failure necessitating longer inter-set rest periods.

Our second hypothesis was only partially supported, as MV_fastest_ decreased with an increase in the number of sets for R1, but not for R3 or R5. Since the external load remained consistent throughout the four sets, a decline in MV_fastest_ serves as an indicator of increased fatigue (i.e., reduced capacity to generate force) at the onset of the set. This is the first study to examine the selective influence of inter-set rest periods on MV_fastest_. A notable discovery is that the behavior of the MNR (an indicator of maximal velocity maintenance capacity) and MV_fastest_ (an indicator of maximal velocity production capacity) differs significantly. For instance, when utilizing COV_0.55_ and the R3 protocol, noticeable reductions in the MNR were observed as the number of sets increased, while no significant decrements were observed for MV_fastest_. These results suggest that the duration of inter-set rest periods, likely in conjunction with other acute RT variables, has distinct effects on both maximal velocity production and muscular endurance. These findings warrant further validation in future studies, as this represents the first investigation specifically focusing on the selective impact on maximal velocity production (performance at the start of the set) and velocity maintenance (performance at the conclusion of the set) capacities. Future studies should also examine whether neural, metabolic, or neuroendocrine mechanisms can explain these findings.

Numerous studies have used relative MVTs (i.e., MVD) as a means to prescribe the number of repetitions during sets not performed to failure (García-Ramos et al., 2021; Pareja-Blanco et al., 2017, 2020a, 2020b; Weakley et al., 2020a, 2020b). It is important to note that all these studies computed MVD using the MV_fastest_ of each specific set as the reference repetition. However, confirming our third hypothesis, in the present study the choice of the reference repetition proved to influence the magnitude of both MVD and MVM. More positive values, reflecting a greater mechanical performance for MVM and a lower fatigue development for MVD, were observed using the MV_fastest_ of each individual set compared to the use of the MV_fastest_ of the first set or from the entire training session. These results demonstrate that in RT sessions characterized by a progressive reduction in MV_fastest_, using relative MVTs (e.g., 20% velocity loss from the MV_fastest_ within the set) for determining the number of repetitions may result in greater levels of fatigue at the end of the set. Therefore, it seems important to consider the interplay between MV_fastest_ and relative MVTs when designing RT programs, as it can influence the resulting fatigue levels experienced by individuals.

The main limitations of this study are related to the specific equipment used and the strength levels of participants. The study focused on the prone bench pull exercise using a Smith machine, potentially limiting its generalizability to individuals who engage in different exercises or perform the bench pull using alternative equipment such as free-weights. Furthermore, research has demonstrated that women (compared to men) and weak men (compared to stronger men) require shorter inter-set rest periods to achieve a specific volume when performing sets to failure during the bench press exercise (Ratamess et al., 2012). While none of the interactions related to the factor of sex reached statistical significance in our study, the limited sample size prevented us from conducting a separate analysis to differentiate between subjects with varying strength levels. Therefore, it is plausible that subjects with different strength levels may respond differently to varying inter-set rest periods. Future research should consider incorporating a wider range of exercises, equipment, and subjects with diverse strength levels to enhance the external validity and applicability of the study's conclusions.

## Conclusions

To ensure consistent maximal performance across multiple sets of the prone bench pull exercise, it is advisable to incorporate longer inter-set rest periods (> 3 min). With the utilization of shorter inter-set rest periods, particularly during sets performed with high levels of fatigue (COV_0.55_), both the MNR and MV_fastest_ decrease with an increasing number of sets. The progressive decline in MV_fastest_, which serves as an indicator of fatigue, holds significant implications for practitioners who rely on relative MVTs (e.g., 20% of velocity loss) to prescribe the number of repetitions. It is worth noting that when employing a fixed relative MVT, unless the MV_fastest_ of the first set (or from the entire training session) is used instead of the MV_fastest_ of each individual set, subjects are likely to experience greater fatigue (i.e., greater force reduction) at the end of the set when the MV_fastest_ is reduced.

## References

[ref1] Akcan, IO, & Olmez, C. How does rest interval duration affect performance? An experiment on high-intensity sprint exercises. Balt J Health Phys Act. 2024;16(1): Article5. 10.29359/BJHPA.16.1.05

[ref2] American College of Sports Medicine. (2009). Progression Models in Resistance Training for Healthy Adults. Medicine & Science in Sports & Exercise, 41(3), 687–708. 19204579 10.1249/MSS.0b013e3181915670

[ref3] Azzeme, M. S. A. M., Tan, K., Sazali, M. H., Japilus, S. J. M., Waqqash, E., & Nadzalan, A. M. (2020). The effects of interset rest duration on performance and muscle activation during resistance training. Journal of Physics: Conference Series, 1529(2), 022025.

[ref4] Bird, S. P., Tarpenning, K. M., & Marino, F. E. (2005). Designing Resistance Training Programmes to Enhance Muscular Fitness. Sports Medicine, 35(10), 841–851. 16180944 10.2165/00007256-200535100-00002

[ref5] Davies, T., Orr, R., Halaki, M., & Hackett, D. (2016). Effect of Training Leading to Repetition Failure on Muscular Strength: A Systematic Review and Meta-Analysis. Sports Medicine, 46(4), 487–502. 26666744 10.1007/s40279-015-0451-3

[ref6] De Salles, B. F., Simão, R., Miranda, F., Da Silva Novaes, J., Lemos, A., & Willardson, J. M. (2009). Rest interval between sets in strength training. Sports Medicine, 39(9), 766–777. 10.2165/11315230-000000000-00000 19691365

[ref7] Erickson, K. I., Hillman, C., Stillman, C. M., Ballard, R. M., Bloodgood, B., Conroy, D. E., Macko, R., Marquez, D. X., Petruzzello, S. J., & Powell, K. E. (2019). Physical Activity, Cognition, and Brain Outcomes: A Review of the 2018 Physical Activity Guidelines. Medicine and Science in Sports and Exercise, 51(6), 1242–1251. 31095081 10.1249/MSS.0000000000001936 PMC6527141

[ref8] Faigenbaum, A. D., & Myer, G. D. (2010). Resistance training among young athletes: safety, efficacy and injury prevention effects. British Journal of Sports Medicine, 44(1), 56–63. 19945973 10.1136/bjsm.2009.068098 PMC3483033

[ref9] García-Ramos, A., Weakley, J., Janicijevic, D., & Jukic, I. (2021). Number of Repetitions Performed Before and After Reaching Velocity Loss Thresholds: First Repetition Versus Fastest Repetition-Mean Velocity Versus Peak Velocity. International Journal of Sports Physiology and Performance, 16(7), 950–957. 33691279 10.1123/ijspp.2020-0629

[ref10] Gonzalez, A. M. (2016). Effect of Interset Rest Interval Length on Resistance Exercise Performance and Muscular Adaptation. Strength & Conditioning Journal, 38(6), 65–68.

[ref11] González-Hernández, J. M., Jimenez-Reyes, P., Janicijevic, D., Tufano, J. J., Marquez, G., & Garcia-Ramos, A. (2023). Effect of different interset rest intervals on mean velocity during the squat and bench press exercises. *Sports Biomechanics*, 22(7), 834–847. 10.1080/14763141.2020.1766102 32567492

[ref12] Grgic, J., Lazinica, B., Mikulic, P., Krieger, J. W., & Schoenfeld, B. J. (2017). The effects of short versus long inter-set rest intervals in resistance training on measures of muscle hypertrophy: A systematic review. European Journal of Sport Science, 17(8), 983–993. 28641044 10.1080/17461391.2017.1340524

[ref13] Grgic, J., Schoenfeld, B. J., Orazem, J., & Sabol, F. (2022). Effects of resistance training performed to repetition failure or non-failure on muscular strength and hypertrophy: A systematic review and meta-analysis. Journal of Sport and Health Science, 11(2), 202–211. 33497853 10.1016/j.jshs.2021.01.007 PMC9068575

[ref14] Grgic, J., Schoenfeld, B. J., Skrepnik, M., Davies, T. B., & Mikulic, P. (2018). Effects of Rest Interval Duration in Resistance Training on Measures of Muscular Strength: A Systematic Review. Sports Medicine, 48(1), 137–151. 28933024 10.1007/s40279-017-0788-x

[ref15] Gutiérrez-Flores, D., Alcaraz, P. E., Cormier, P., Martínez-Serrano, A., Freitas, T. T. (2024). Do Activities Performed within the Intra-Contrast Rest Interval Affect Neuromuscular Performance during Complex-Contrast Training Protocols?. Journal of Human Kinetics, 91, 33–46. 10.5114/jhk/184168 38689590 PMC11057618

[ref16] Jiménez-Alonso, A., García-Ramos, A., Cepero, M., Miras-Moreno, S., Rojas, F. J., & Pérez-Castilla, A. (2022). Effect of Augmented Feedback on Velocity Performance During Strength-Oriented and Power-Oriented Resistance Training Sessions. Journal of Strength and Conditioning Research, 36(6), 1511–1517. 32639379 10.1519/JSC.0000000000003705

[ref17] Jukic, I., Castilla, A. P., Ramos, A. G., Van Hooren, B., McGuigan, M. R., & Helms, E. R. (2023). The Acute and Chronic Effects of Implementing Velocity Loss Thresholds During Resistance Training: A Systematic Review, Meta-Analysis, and Critical Evaluation of the Literature. Sports Medicine, 53(1), 177–214. 36178597 10.1007/s40279-022-01754-4 PMC9807551

[ref18] Jukic, I., García-Ramos, A., & Tufano, J. J. (2023). Velocity-Based Resistance Training Monitoring: Influence of Lifting Straps, Reference Repetitions, and Variable Selection in Resistance-Trained Men. Sports Health: A Multidisciplinary Approach, 15(3), 333–341. 10.1177/19417381221095073 PMC10170227 35587704

[ref19] Kraemer, W. J., & Ratamess, N. A. (2004). Fundamentals of Resistance Training: Progression and Exercise Prescription. Medicine & Science in Sports & Exercise, 36(4), 674–688. 15064596 10.1249/01.mss.0000121945.36635.61

[ref20] Kraemer, W. J., & Ratamess, N. A. (2005). Hormonal Responses and Adaptations to Resistance Exercise and Training. Sports Medicine, 35(4), 339–361. 15831061 10.2165/00007256-200535040-00004

[ref21] Lopez, P., Radaelli, R., Taaffe, D. R., Newton, R. U., Galvão, D. A., Trajano, G. S., Teodoro, J. L., Kraemer, W. J., Häkkinen, K., & Pinto, R. S. (2021). Resistance Training Load Effects on Muscle Hypertrophy and Strength Gain: Systematic Review and Network Meta-analysis. Medicine and Science in Sports and Exercise, 53(6), 1206–1216. 33433148 10.1249/MSS.0000000000002585 PMC8126497

[ref22] Miras-Moreno, S., Pérez-Castilla, A., & García-Ramos, A. (2022). Lifting Velocity as a Predictor of the Maximum Number of Repetitions That Can Be Performed to Failure During the Prone Bench Pull Exercise. International Journal of Sports Physiology and Performance, 17(8), 1213–1221. 35700976 10.1123/ijspp.2021-0534

[ref23] Pareja-Blanco, F., Alcazar, J., Cornejo-Daza, P. J., Sánchez-Valdepeñas, J., Rodriguez-Lopez, C., Hidalgo-de Mora, J., Sánchez-Moreno, M., Bachero-Mena, B., Alegre, L. M., & Ortega-Becerra, M. (2020a). Effects of velocity loss in the bench press exercise on strength gains, neuromuscular adaptations, and muscle hypertrophy. *Scandinavian Journal of Medicine & Science in Sports*, 30(11), 2154–2166. 32681665 10.1111/sms.13775

[ref24] Pareja-Blanco, F., Alcazar, J., Sánchez-Valdepeñas, J., Cornejo-Daza, P. J., Piqueras-Sanchiz, F., Mora-Vela, R., Sánchez-Moreno, M., Bachero-Mena, B., Ortega-Becerra, M., & Alegre, L. M. (2020b). Velocity Loss as a Critical Variable Determining the Adaptations to Strength Training. Medicine and Science in Sports and Exercise, 52(8), 1752–1762. 32049887 10.1249/MSS.0000000000002295

[ref25] Pareja-Blanco, F., Rodríguez-Rosell, D., Sánchez-Medina, L., Sanchis-Moysi, J., Dorado, C., Mora-Custodio, R., Yáñez-García, J. M., Morales-Alamo, D., Pérez-Suárez, I., Calbet, J. A. L., & González-Badillo, J. J. (2017). Effects of velocity loss during resistance training on athletic performance, strength gains and muscle adaptations. Scandinavian Journal of Medicine & Science in Sports, 27(7), 724–735. 27038416 10.1111/sms.12678

[ref26] Pérez-Castilla, A., Miras-Moreno, S., Weakley, J., & García-Ramos, A. (2023). Relationship Between the Number of Repetitions in Reserve and Lifting Velocity During the Prone Bench Pull Exercise: An Alternative Approach to Control Proximity-to-Failure. Journal of Strength and Conditioning Research, 37(8), 1551–1558. 36662153 10.1519/JSC.0000000000004448

[ref27] Ratamess, N. A., Faigenbaum, A. D., Chiarello, C. M., Ross, R. E., Kang, J., Sacco, A. J., & Hoffman, J. R. (2012). The effects of rest interval length on acute bench press performance: the influence of gender and muscle strength. *Journal of Strength and Conditioning Research*, 26(7), 1817–1826. 22561970 10.1519/JSC.0b013e31825bb492

[ref28] Ratamess, N. A., Falvo, M. J., Mangine, G. T., Hoffman, J. R., Faigenbaum, A. D., & Kang, J. (2007). The effect of rest interval length on metabolic responses to the bench press exercise. *European Journal of Applied Physiology*, 100(1), 1–17. 17237951 10.1007/s00421-007-0394-y

[ref29] Sánchez-Medina, L., & González-Badillo, J. J. (2011). Velocity loss as an indicator of neuromuscular fatigue during resistance training. Medicine and Science in Sports and Exercise, 43(9), 1725–1734. 21311352 10.1249/MSS.0b013e318213f880

[ref30] Tsoukos, A., Krzysztofik, M., Wilk, M., Zajac, A., Panagiotopoulos, M. G., Psarras, I. ... Bogdanis, G. C. (2024). Fatigue and Metabolic Responses during Repeated Sets of Bench Press Exercise to Exhaustion at Different Ranges of Motion. *Journal of Human Kinetics*, 91, 61–76. 10.5114/jhk/185524 38689577 PMC11057609

[ref31] Tufano, J. J., Conlon, J. A., Nimphius, S., Brown, L. E., Seitz, L. B., Williamson, B. D., & Gregory Haff, G. (2016). Maintenance of Velocity and Power With Cluster Sets During High-Volume Back Squats. International Journal of Sports Physiology and Performance, 11(7), 885–892. 26791936 10.1123/ijspp.2015-0602

[ref32] Weakley, J., McLaren, S., Ramirez-Lopez, C., García-Ramos, A., Dalton-Barron, N., Banyard, H., Mann, B., Weaving, D., & Jones, B. (2020a). Application of velocity loss thresholds during free-weight resistance training: Responses and reproducibility of perceptual, metabolic, and neuromuscular outcomes. Journal of Sports Sciences, 38(5), 477–485. 31868099 10.1080/02640414.2019.1706831

[ref33] Weakley, J., Morrison, M., García-Ramos, A., Johnston, R., James, L., & Cole, M. H. (2021). The Validity and Reliability of Commercially Available Resistance Training Monitoring Devices: A Systematic Review. Sports Medicine, 51(3), 443–502. 33475985 10.1007/s40279-020-01382-w PMC7900050

[ref34] Weakley, J., Ramirez-Lopez, C., McLaren, S., Dalton-Barron, N., Weaving, D., Jones, B., Till, K., & Banyard, H. (2020b). The Effects of 10%, 20%, and 30% Velocity Loss Thresholds on Kinetic, Kinematic, and Repetition Characteristics During the Barbell Back Squat. International Journal of Sports Physiology and Performance, 15(2), 180–188. 31094251 10.1123/ijspp.2018-1008

[ref35] Weakley, J., Schoenfeld, B. J., Ljungberg, J., Halson, S. L., & Phillips, S. M. (2023). Physiological Responses and Adaptations to Lower Load Resistance Training: Implications for Health and Performance. *Sports Medicine*, 9(1), 28. 10.1186/s40798-023-00578-4 PMC10182225 37171517

[ref36] Wilk, M., Golas, A., Stastny, P., Nawrocka, M., Krzysztofik, M., & Zajac, A. (2018). Does Tempo of Resistance Exercise Impact Training Volume? Journal of Human Kinetics, 62(1), 241–250. 29922395 10.2478/hukin-2018-0034 PMC6006544

[ref37] Wilk, M., Krzysztofik, M., Gepfert, M., Poprzecki, S., Golas, A., & Maszczyk, A. (2018a). Technical and Training Related Aspects of Resistance Training Using Blood Flow Restriction in Competitive Sport-A Review. Journal of Human Kinetics, 65(4), 249–260. 30687436 10.2478/hukin-2018-0101 PMC6341949

